# Expérience Marocaine sur les goitres négligés en mission humanitaire en Guinée Bissau: à propos de quatre observations

**DOI:** 10.11604/pamj.2015.22.72.7935

**Published:** 2015-09-29

**Authors:** Hicham Laraqui, Khalid Sair

**Affiliations:** 1 Service de Chirurgie Viscérale, Hôpital Militaire d'Instruction Mohammed V Rabat, Maroc

**Keywords:** Tumeurs historiques, retard diagnostique, niveau socio-économique, historical tumors, delayed diagnosis, socioeconomic level

## Abstract

La mission humanitaire de notre hôpital militaire médico-chirurgicale en Guinée Bissau avait pour but le rapprochement des soins à la population. Les pathologies rencontrées étaient le plus souvent négligées. Nous rapportons quatre observations de goitres négligés sur une durée de trois mois allant de Mai 2015 à Aout 2015. Nous discuterons les problèmes de pathologies négligées en rapport essentiellement avec le niveau socio-économique bas et l'insuffisance en moyens diagnostiques et thérapeutiques et en personnel de soins qualifiés.

## Introduction

La mission humanitaire de l'hôpital militaire médico-chirurgicale en Guinée Bissau avait pour but le rapprochement des soins à la population. Les pathologies rencontrées étaient le plus souvent négligées. Nous rapportons quatre observations de goitres négligés sur une durée de trois mois allant de Mai 2015 à Aout 2015. L'objectif de notre travail est de mettre le point sur les difficultés rencontrées pour la prise en charge des patients dans un tel contexte.

## Patients observations

**Figure 1:**
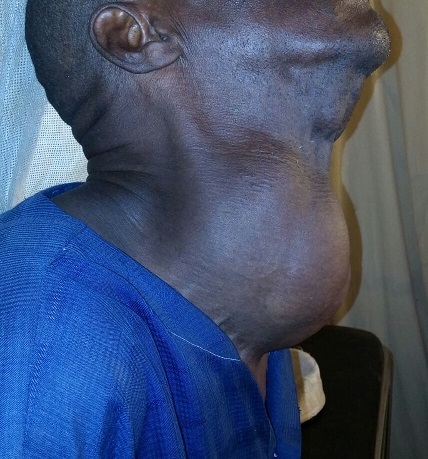
Goitre volumineux homogène mobile à la déglutition de consistance molle (observation 1)

**Figure 2:**
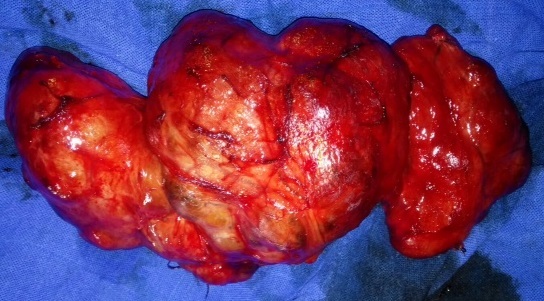
Pièce de thyroïdectomie totale (observation 1)

**Observation 1**: il s'agissait d'un homme de 65 ans, sans antécédents notables qui a consulté dans notre unité pour un goitre évoluant 30 ans auparavant. L’évolution clinique était marquée par l'augmentation du volume avec signes de gêne fonctionnelle sans notion de dysthyroidie clinique. L'examen clinique retrouvait un goitre volumineux homogène mobile à la déglutition de consistance molle sans adénopathie locorégionale (Figure 1). Le pouls était estimé à 80 battements/minute et la tension artérielle à 120/80. Un bilan biologique standard a été réalisé et qui est revenu sans anomalie. Le bilan biologique thyroïdien et les examens radiologiques n'ont pas été réalisé par défaut de moyen mis à part une radiographie des parties molles du cou montrant une déviation trachéale sans caractère plongeant du goitre. Une thyroïdectomie totale a été réalisée (Figure 2). L’étude anatomopathologique n’était pas disponible. Les suites opératoires ont été simples, le malade a quitté l'hôpital au bout de trois jours sous traitement oral substitutif et il a été confié à une structure locale pour le suivi.

**Figure 3:**
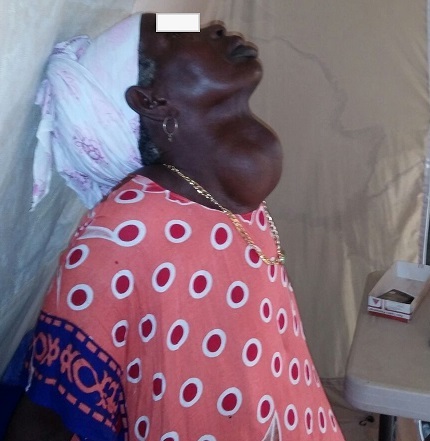
Goitre volumineux prédominant sur les deux lobes mobile sans caractère d'induration (observation 2)

**Observation 2**: il s'agissait d'une patiente de 55 ans, sans antécédents notables, qui vient dans notre consultation pour un goitre évoluant depuis 15 ans sans aucun symptome. L'examen clinique trouvait un goitre volumineux prédominant sur les deux lobes mobile sans caractère d'induration et sans adénopathie (Figure 3). Le pouls était à 75 pulsations/min. la radiographie cervicale standard avait mis en évidence une déviation trachéale sans caractère plongeant. Une thyroïdectomie totale a été réalisée. Les suites opératoires ont été simples, la malade est sortie sous traitement oral substitutif et confié à une équipe locale pour le suivi.

**Figure 4:**
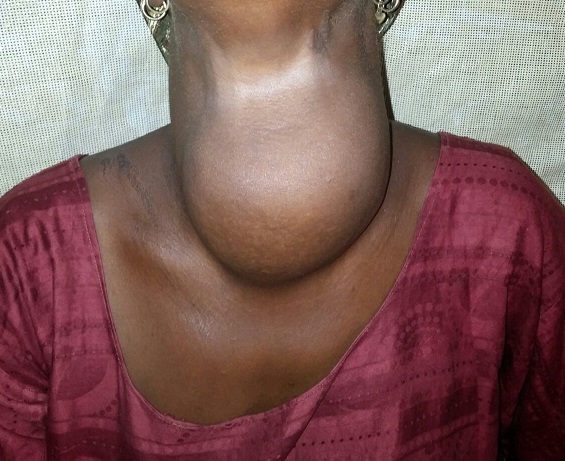
Goitre à prédominance gauche mobile à la déglutition (Observation 3)

**Observation 3**: il s'agissait d'une patiente de 35 ans, sans antécédents notables, qui avait consulté pour goitre évoluant 10 ans auparavant sans signe clinque de dysthyroidie retentissant sur la vie conjugale de la patiente. L'examen clinique trouvait un goitre à prédominance gauche mobile à la déglutition sans dysphonie ni dysphagie (Figure 4). Les aires ganglionnaires étaient libres. Le pouls était à 80 battements/minute. Le bilan biologique standard est revenu normal. La radiographie des parties molles du cou a mis en évidence une déviation trachéale sans calcifications. Le geste chirurgical a consisté en une isthmolobectomie gauche avec vérification par échographie peropératoire l'absence de nodule sur le lobe restant. Les suites opératoires étaient simples, la malade a quitté l'hôpital trois jours après sans traitement substitutif.

**Figure 5:**
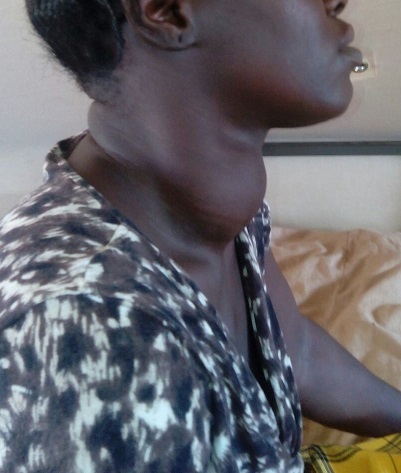
Goitre à prédominance droite n'arrivant à limiter son bord inférieur (observation 4)

**Observation 4**: il s'agissait d'une patiente de 25 ans, sans antécédents notables, qui a consulté pour un goitre évoluant depuis 10 ans avec un légèr gène respiratoire. Sans signe clinique de dysthyroidie. L'examen clinique retrouvait un goitre à prédominance droite n'arrivant à limiter son bord inférieur (Figure 5). Les aires ganglionnaires étaient libres. Le pouls était à 75 battements/minute. La radiographie cervicale n'a pas été faite par panne de l'appareil. Le geste chirurgical a consisté en une isthmolobectomie droite notant le caractère plongeant de la thyroïde accouché par la même incision malgré le volume important. Les suites opératoires ont été simples, la malade a quitté l'hôpital six jours après sans traitement substitutif.

## Discussion

La prise en charge des patients de la guinée Bissau dans l'hôpital militaire médico-chirurgical mobil marocain, nous a fait réfléchir sur la question liée au retard de consultation et les facteurs associés qui ont poussé ces patients à négliger leur pathologie ce qui expose à des complications de morbidité sans oublier le coté pshychosocial. Notre expérience de consultation de pathologie thyroïdienne étalée sur une période de trois mois nous a permis de voir 50 patients porteurs de cette pathologie dont 20 en euthyroidie clinique dont nous rapportons 04 cas de goitres négligés de dimensions importantes. Le retard à la consultation s'explique d'une part par l'absence de formation hospitalière, de médecins spécialistes et d'infrastructure pour les laboratoires d'analyse médicale et d'anatomie pathologique. D'autre part, l'isolement social, le niveau socio-économique bas et l'absence de couverture sociale adéquate empêchent ces patients d'aller solliciter des soins dans les pays de voisinage. Deux études prospectives publiées en 1993 et 1995 portant sur des patients atteints de tumeurs cutanées historiques. La négligence était causé en partie par un isolement social et un niveau socioé-conomique bas [1, 2]. Notre présence au sein de cette population via notre hôpital, a permis d'offrir une proximité de soins médico-chirurgicale. Il est à noté également que l'indication chirurgicale s'est basé souvent sur les données cliniques, quelques bilans biologiques standards, la radiographie pulmonaire et ECG excluant ainsi les patients en dysthyroidie clinique d'autant plus la durée courte de notre mission ne nous a pas permis de préparer les autres patients pour un acte chirurgical dans les conditions biologiques optimales. De ce fait, on a pu opérer 20 patients atteints de goitres sur une durée de 03 mois. Les actes chirurgicaux réalisés ont consisté en des isthmolobectomies et des thyroïdectomies totales. Les difficultés chirurgicales rencontrées étaient en rapport avec le volume et le caractère plongeant de certains goitres comme fut le cas de notre quatrième observation. Dans la littérature, les pathologies négligées posent souvent le problème du traitement, les gestes chirurgicaux sont plus complexes [3]. Toutefois, nous n'avons pas noté des cas de morbidité péri-opératoires. Par ailleurs, dans notre expérience nous n'avons pas pu évaluer le pourcentage de transformation maligne vu l'absence d'un laboratoire d'anatomie pathologique sur site ni dans le pays.

## Conclusion

L'intérêt de ce travail est de projeter la lumière sur un problème de pathologies négligées en rapport essentiellement avec le niveau socio-économique bas et l'absence d'infrastructure et de personnel médicales et paramédicales spécialisés. Après notre mission, nous recommandons pour pallier aux difficultés de retard de prise en charge de ces patients en Guinée Bissau ou dans d'autres pays similaires de multiplier ce genre de mission humanitaire en l'attente de former du personnel médical et paramédical et d'aménager l'infrastructure adéquate.
